# Relative Entropy and Optimization-Driven Coarse-Graining Methods in VOTCA

**DOI:** 10.1371/journal.pone.0131754

**Published:** 2015-07-20

**Authors:** S. Y. Mashayak, Mara N. Jochum, Konstantin Koschke, N. R. Aluru, Victor Rühle, Christoph Junghans

**Affiliations:** 1 Department of Mechanical Science and Engineering, Beckman Institute for Advanced Science and Technology, University of Illinois at Urbana-Champaign, Urbana, Illinois, 61801, United States of America; 2 Max Planck Institute for Polymer Research, Ackermannweg 10, 55128 Mainz, Germany; 3 Department of Chemistry, University of Cambridge, Cambridge, United Kingdom; 4 Computer, Computational, and Statistical Sciences Division, Los Alamos National Laboratory, Los Alamos, NM 87545, United States of America; Hong Kong University of Science and Technology, HONG KONG

## Abstract

We discuss recent advances of the VOTCA package for systematic coarse-graining. Two methods have been implemented, namely the downhill simplex optimization and the relative entropy minimization. We illustrate the new methods by coarse-graining SPC/E bulk water and more complex water-methanol mixture systems. The CG potentials obtained from both methods are then evaluated by comparing the pair distributions from the coarse-grained to the reference atomistic simulations. In addition to the newly implemented methods, we have also added a parallel analysis framework to improve the computational efficiency of the coarse-graining process.

## 1 Introduction

In recent years, coarse-grained simulations have become an important tool for investigating systems on larger time- and length-scales [1–6]. Here, we focus on the bottom-up approaches of coarse-graining (CG), which create a systematic link between two particle based descriptions of a given system by building a lower resolution model based on a reference higher resolution model.

In a coarse-grained representation, several atoms are grouped into a single CG unit and the effective interactions between them are determined. Such coarse-graining leads to a significant speed-up in the molecular dynamics (MD) simulations due to i) a reduced number of degrees of freedom and hence, the number of interactions to compute, ii) smoother interaction potentials among CG particles that enable larger time steps, and most importantly, iii) a speedup in the intrinsic dynamics of the system which leads to faster diffusion and thus, shorter equilibration times [7].

Although coarse-grained simulations allow one to reach longer time- and length-scales, they also, by definition, lose some information from the underlying reference system. For instance, when replacing a water molecule by a single sphere, one loses information about the molecule’s orientation. Generally, CG systems are limited in their representability and transferability, i.e., they cannot simultaneously reproduce all of the properties of interest and they may not be applicable for thermodynamic states far away from the one at which the system was parametrized [8, 9].

There exist, however, several coarse-graining techniques of varying complexities and accuracies for modeling CG systems and optimizing their interactions. These range from structure-based methods, such as Boltzmann inversion, iterative Boltzmann inversion (IBI) [10], inverse monte carlo (IMC) [11], and relative entropy [12], to force-based methods like force matching (FM) [13, 14]. Structure-based methods target (multi-body) distribution functions and use the relation between the distribution functions and the potential of mean force (PMF) [10, 14] to derive effective CG interactions. In contrast, the FM method tries to reproduce forces on CG sites [14]. Detailed descriptions about the various aspects and methods for determining CG potentials can be found in ref. [1, 3, 14–16].

Given such a wide range of methods of varying complexity and properties that they target, the question arises as which method is the most suitable for a specific system in a desired application. This can be best answered by a comparative analysis of the CG potentials obtained from different methods. To facilitate the determination of these potentials and to allow for a direct comparison of the methods, the Versatile Object-Oriented Toolkit for Coarse-graining Applications (VOTCA) was developed by Rühle et. al [16], which is available under an open source software license.

In the first release of VOTCA, Boltzmann inversion, IBI, IMC, and FM had been implemented. In ref. [16], the VOTCA package was used to compare IBI, IMC, and FM by coarse-graining four test systems, namely SPC/E water, methanol, liquid propane, and a single chain of hexane. For example, it was observed that the IMC update function is more efficient than that of IBI method. IMC, however, requires significantly more statistics, and hence longer trajectories, and can be sensitive to finite size effects. On the other side, FM can have representability issues if the set of coarse-grained interactions is incomplete. For such cases, three hybrid schemes to combine FM for non-bonded and the Boltzmann inversion for bonded interactions have been discussed in ref. [17] and applied to liquid hexane.

Recently, we added two new methods to the VOTCA package, namely the simplex method [18] and relative entropy method [12].

The simplex algorithm is a general optimization method, which can be used to optimize coarse-grained interactions. As such, it is difficult to target properties other than structure, e.g. thermodynamic quantities. Nevertheless, extensions for pressure correction [10] or the Kirkwood-Buff integral (KBI) [19] have been proposed but lack in a sound theoretical basis. However, structure-based methods, pressure correction, or KBI, are based on the relation between the CG interactions and target properties. By comparison, the simplex algorithm makes no *a priori* assumptions about the relation between the effective interactions and the target properties but instead requires the definition of an objective function which allows to fit arbitrary properties using a given functional form for the potentials. We call this approach targeted coarse-graining. In that sense, structure-based methods could be categorized as a subclass of targeted coarse-graining methods which target structural quantities and use a specialized optimizers like IMC or IBI.

Relative entropy is a method which quantifies the extent of the configurational phase-space overlap between two molecular ensembles [20]. It can be used as a measure of the discrepancies between various properties of the CG system’s and the target all-atom (AA) ensemble. It has been shown by Shell S. [12] that one can minimize the relative entropy metric between the model CG system and the target AA system to optimize CG potential parameters such that the CG ensemble would mimic the target AA ensemble.

The main objective of this paper is to describe and demonstrate the two newly implemented methods in VOTCA. In the sections 2.1 and 2.2, we describe the formulations of the simplex-based and the relative entropy-based methods, respectively. Implementation details of the simplex and relative entropy methods are given in the sections 3.1 and 3.2, respectively. In the sections 4.1 and 4.2, we illustrate the simplex and relative entropy methods for a three-site SPC/E bulk water and a more complex water-methanol bulk mixture. To evaluate the accuracy of the CG potentials, we then compare the two-body distribution functions obtained from the CG MD system (CG-MD) with the reference AA MD system (AA-MD) simulations. In addition to these new methods, we have also made several improvements and other additions to VOTCA as described in the sections 3.4 and 3.5. For example, we have added a parallel analysis framework, which improves the performance of VOTCA by allowing it to utilize multi-core CPUs. These performance improvements are discussed in the section 3.4.

## 2 Methods

### 2.1 Downhill simplex algorithm

The downhill simplex algorithm [18] is a derivative-free optimization procedure designed to minimize an objective (or penalty) function, *y*(*x*_1_, *x*_2_, …, *x*_*n*_), with *n* degrees of freedom.

In the algorithm, the current state is described by *n*+1 vertices ***p***_1_, ***p***_2_, …, ***p***_*n*+1_, with their corresponding penalty values from the objective function, which make up the geometric shape of a polytope. This polytope can then be transformed successively using basic operations such as a reflection, an expansion, a contraction, or a reduction in order to move towards the global minimum, i.e., minimize the objective function. The nature of progression of the algorithm depends on the combination of these transformations. In the following notation, we assume that the vertices at each step *i* are sorted according to their penalty values in ascending order.

The first transformation performed on the initial polytope is a *reflection*, in which the vertex with the highest objective value, ***p***_*n*+1_, is reflected through the centroid of all remaining points, $\bar{p}$, such that

$$\begin{array}{c} p_{r}=\bar{p}+\alpha (\bar{p}-p_{n+1})\;,\;\alpha >0\;. \end{array}$$ (2.1)

Here, ***p***_*r*_ is the reflected point and *α* is the reflection coefficient. This transformation preserves the volume of the simplex. If the penalty of the reflected point, *y*_*r*_, is lower than the best value, i.e., *y*_*r*_ < *y*_1_, the algorithm tries to increase the step size along the line of the reflection. This is known as an *expansion*,

$$\begin{array}{c} p_{e}=\bar{p}+\gamma (\bar{p}-p_{n+1})\;,\;\gamma >1\;, \end{array}$$ (2.2)

where ***p***_*e*_ is the expanded point and *γ* is the expansion coefficient, which is the distance from ***p***_*r*_ through the centroid, $\bar{p}$, to ***p***_*e*_. In contrast, if the value of the reflected point is worse than the highest point, i.e., *y*_*r*_ > *y*_*n*+1_, the move is too large. Thus, a *contraction* is performed to reduce the step size and find a point which decreases the objective function, where

$$\begin{array}{c} p_{c}=\bar{p}+\rho (\bar{p}-p_{n+1})\;,\;0>\rho >1\;. \end{array}$$ (2.3)

Here, ***p***_*c*_ is the contracted point and *ρ* is the contraction coefficient. If no improvement can be achieved upon contraction, i.e., *y*_*c*_ > *y*_*n*+1_, a *reduction* is performed. This happens in situations close to a minimum by contracting the whole simplex around the lowest point, ***p***_1_, such that

$$\begin{array}{c} p_{i}\to \sigma (p_{i}+p_{1})\;, \end{array}$$ (2.4)

where *σ* is the reduction coefficient. Subsequently, the procedure is restarted with a reflection. In all other cases, the highest point, ***p***_*n*+1_, is first replaced by the newly reflected, expanded, or contracted point, all points are resorted according to their penalty values, and the procedure is then resumed.

The above described algorithm is applicable to a wide range of applications. In the context of coarse-graining, the evaluation of the objective function involves performing a coarse-grained simulation with a given set of parameters and subsequently analyzing its trajectory to compute the properties of interest. Here, the objective function calculates a single value, which is a cumulative measure of how well the target properties are reproduced. Hence, the algorithm constitutes a coarse-graining tool to find the optimal coarse-grained interaction parameters for nearly any measurable simulation property, even the optimal mapping for the CG model itself.

One of the benefits of the downhill simplex algorithm is that it does not require any derivatives of the objective function which are often difficult to obtain. However, problems with convergence may arise when poor initial guesses are provided which yield high penalty values or if the objective function contains too many parameters, such as *n* > 10. Furthermore, since the objective function is a cumulative measure of the properties of interest, providing an appropriate form which accounts for each of these equally is often challenging.

In this work, we set *α* = 1, *γ* = 2, and *ρ*, *σ* = 0.5. However, as the performance of the simplex algorithm heavily depends on the type of system and the properties of interest studied, adapting these transformation coefficients may lead to a better performance than reported here.

### 2.2 Relative entropy

Relative entropy, *S*_rel_, is defined as [12]

$$\begin{array}{c} S_{\text{rel}}=\sum_{i}p_{\text{AA}}\left(r_{i}\right)\ln(\frac{p_{\text{AA}}\left(r_{i}\right)}{p_{\text{CG}}(M(r_{i}))})+{\left\langle S_{\text{map}}\right\rangle }_{\text{AA}}\;, \end{array}$$ (2.5)

where the sum is over all the configurations of the reference AA system, *r* = {*r*_*i*_}(*i* = 1, 2, …), *M* is the mapping operation to generate a corresponding CG configuration, *R*_*I*_, from a AA configuration, *r*_*i*_, i.e., *R*_*I*_ = *M*(*r*_*i*_), *p*_AA_ and *p*_CG_ are the configurational probabilities based on the AA and CG potentials, respectively, and ⟨*S*_map_⟩_AA_ is the mapping entropy due to the average degeneracy of AA configurations mapping to the same CG configuration, given by

$$\begin{array}{c} S_{\text{map}}\left(R_{I}\right)=\ln\sum_{i}\delta_{R_{I},M\left(r_{i}\right)}\;, \end{array}$$ (2.6)

where *δ* is the Kronecker delta function. From Eq (2.6), it can be shown that the mapping entropy, ⟨*S*_map_⟩_AA_, does not depend on the CG interactions, but instead it is a unique function of the mapping operation, *M*, and the AA configurational weights. The relative entropy is a metric borrowed from the field of information theory, which quantifies the extent of the configurational phase-space overlap between two molecular ensembles [20]. The log-likelihood based derivation of the relative entropy for molecular systems, as defined in Eq (2.5), is given in ref. [12]. Physically, *S*_rel_ can be interpreted as the log probability that one test configuration of the model CG ensemble is representative of the target AA ensemble, and when the likelihood is a maximum, *S*_rel_ is at a minimum. Hence, the numerical minimization of *S*_rel_ with respect to the parameters of the CG model can be used to optimize the CG model.

Comparisons between relative entropy and other coarse-graining methods are made in ref. [14] and [15]. Chaimovich and Shell [15] have shown that for certain CG models relative entropy minimization produces the same CG potentials as other methods, e.g., it is equivalent to the IBI when CG interactions are modeled using finely tabulated pair additive potentials, and to the FM when a CG model is based on *N*–body interactions, where *N* is the number of degrees of freedom in the CG model. However, there are some advantages of using relative entropy based coarse-graining. Relative entropy method allows to use analytical function forms for CG potentials, which are desired in theoretical treatments, such as parametric study of CG potentials, whereas, methods, like IBI, use tabulated potentials. Recently Lyubartsev et. al [21] have shows how to use IMC with an analytical function form, too. BI, IBI, and IMC methods are based on pair correlations and hence, they are only useful to optimize 2-body CG potentials, whereas, relative entropy uses more generic metric which offers more flexibility in modeling CG interactions and not only 2-body, but also 3-body and N-body CG potentials can be optimized. In addition to the CG potential optimization, the relative entropy metric can also be used to optimize an AA to CG mapping operator.

In a canonical ensemble, substituting canonical configurational probabilities into Eq (2.5), the relative entropy simplifies to

$$\begin{array}{c} S_{\text{rel}}=\beta {\left\langle U_{\text{CG}}-U_{\text{AA}}\right\rangle }_{\text{AA}}-\beta (A_{\text{CG}}-A_{\text{AA}})+{\left\langle S_{\text{map}}\right\rangle }_{\text{AA}}\;, \end{array}$$ (2.7)

where *β* = 1/*k*_B_ *T*, *k*_B_ is the Boltzmann constant, *T* is the temperature, *U*_CG_ and *U*_AA_ are the total potential energies from the CG and AA potentials, respectively, *A*_CG_ and *A*_AA_ are the configurational part of the Helmholtz free energies from the CG and AA potentials, respectively, and all the averages are computed in the reference AA ensemble.

Consider a model CG system defined by the CG potentials between various CG sites such that the CG potentials depend on the parameters **λ** = {*λ*_1_, *λ*_2_, …*λ*_*n*_}. As described above, in the relative entropy based coarse-graining, the CG potential parameters, **λ**, are optimized by the relative entropy minimization.

In this work, we use the Newton-Raphson strategy for the relative entropy minimization described in ref. [15]. In this strategy, the CG potential parameters, **λ**, are refined iteratively as

$$\begin{array}{c} \lambda^{k+1}=\lambda^{k}-\chi {\text{H}}^{-1}\cdot \nabla_{\lambda }S_{\text{rel}}\;, \end{array}$$ (2.8)

where *k* is the iteration index, *χ* ∈ (0…1) is the relaxation parameter that can be adjusted to ensure convergence, ∇_*λ*_ *S*_rel_ is the vector of the first derivatives of *S*_rel_ with respect to **λ**, which can be computed from Eq (2.7) as

$$\begin{array}{c} \nabla_{\lambda }S_{\text{rel}}=\beta {\left\langle \frac{\partial U_{\text{CG}}}{\partial \lambda }\right\rangle }_{\text{AA}}-\beta {\left\langle \frac{\partial U_{\text{CG}}}{\partial \lambda }\right\rangle }_{\text{CG}}\;, \end{array}$$ (2.9)

and **H** is the Hessian matrix of *S*_rel_ given by

$$\begin{array}{ccc} {\text{H}}_{ij} & = & \beta {\left\langle \frac{\partial^{2}U_{\text{CG}}}{\partial \lambda_{i}\partial \lambda_{j}}\right\rangle }_{\text{AA}}-\beta {\left\langle \frac{\partial^{2}U_{\text{CG}}}{\partial \lambda_{i}\partial \lambda_{j}}\right\rangle }_{\text{CG}}\\ & & +\beta^{2}{\left\langle \frac{\partial U_{\text{CG}}}{\partial \lambda_{i}}\frac{\partial U_{\text{CG}}}{\partial \lambda_{j}}\right\rangle }_{\text{CG}}\\ & & -\beta^{2}{\left\langle \frac{\partial U_{\text{CG}}}{\partial \lambda_{i}}\right\rangle }_{\text{CG}}{\left\langle \frac{\partial U_{\text{CG}}}{\partial \lambda_{j}}\right\rangle }_{\text{CG}}. \end{array}$$ (2.10)

To compute ∇_*λ*_ *S*_rel_ and **H** from Eqs (2.9) and (2.10), we need average CG energy derivatives in the AA and CG ensembles. For the averages in the AA ensemble, first a single AA system simulation can be performed and its AA configurations can be saved, then the average CG energy derivatives can be computed by processing the mapped CG configurations of the saved AA configurations using the CG potentials at each iteration. For the averages in the CG ensemble, since the CG ensemble changes with the CG parameters, **λ**, a short CG simulation can be performed at each iteration to generate corresponding CG configurations. An alternative approach, which does not require a CG simulation at every iteration, to obtain the CG ensemble averages is to reweight the initial CG configurations obtained from **λ** ^0^ [15]. In this work, we implemented the first approach based on performing a short CG simulation at every iteration.

In the case of a CG model, in which CG interactions are modeled by a two-body pair potential, *u*_CG_, between CG sites, the ensemble averages of the CG energy derivatives can be computed as

$$\begin{array}{ccc} {\left\langle {\left(\frac{\partial^{a}U_{\text{CG}}}{\partial \lambda^{a}}\right)}^{b}\right\rangle }_{\text{AA}} & = & {\left\langle {\left(\sum_{i<j}\frac{\partial^{a}u_{\text{CG}}\left(r_{ij}\right)}{\partial \lambda^{a}}\right)}^{b}\right\rangle }_{\text{AA}}\\ {\left\langle {\left(\frac{\partial^{a}U_{\text{CG}}}{\partial \lambda^{a}}\right)}^{b}\right\rangle }_{\text{CG}} & = & {\left\langle {\left(\sum_{i<j}\frac{\partial^{a}u_{\text{CG}}\left(r_{ij}\right)}{\partial \lambda^{a}}\right)}^{b}\right\rangle }_{\text{CG}}\;, \end{array}$$ (2.11)

where the sum is performed over all the CG site pairs (*i*, *j*), *a* stands for the 1^st^, 2^nd^, … derivatives and *b* stands for the different powers, i.e., *b* = 1, 2, ….

## 3 Implementation

### 3.1 Simplex

To be versatile, we have implemented a generic optimization engine embedded in the existing iterative script framework of VOTCA. This optimizer uses a state file to communicate the potential energy parameters for the next MD simulation to be performed. Here, potential energy tables are generated from these parameters which are then used in the simulation. After the simulation is complete, it can be analyzed to evaluate the objective function and again communicate back the next set of parameters to the optimizer via the state file.

The state-based approach described above allows one to easily swap different optimization algorithms. In this work, we have implemented the downhill simplex algorithm (in Perl) and an interface to the existing covariance matrix adaptation (CMA) evolution strategy optimizer [22] (written in Python). CMA is reported to have a better convergence if a large number of parameters are to be optimized.

### 3.2 Relative entropy

In the VOTCA package, we implemented the relative entropy-based coarse-graining method using the iterative workflow framework described in ref. [16]. Required inputs are the pair distributions of the CG sites in the reference AA ensemble, the initial guess for the CG potential parameters, the CG ensemble simulation set up files, and the option file describing the modeling options for the CG interactions. Furthermore, the user can provide the Newton-Raphson iteration parameters, such as the relaxation parameter, *χ*, the convergence check criteria, etc.

To describe the CG potentials, we use the uniform cubic B-spline (CBSPL) form given by

$$\begin{array}{c} u_{\text{CBSPL}}\left(r\right)=[\begin{array}{cccc} 1 & t & t^{2} & t^{3} \end{array}]\frac{1}{6}[\begin{array}{cccc} 1 & 4 & 1 & 0\\ -3 & 0 & 3 & 0\\ 3 & -6 & 3 & 0\\ -1 & 3 & -3 & 1 \end{array}][\begin{array}{c} c_{k}\\ c_{k+1}\\ c_{k+2}\\ c_{k+3} \end{array}], \end{array}$$ (3.1)

where {*c*_0_, *c*_1_, *c*_2_, …, *c*_*m*_} are the spline knot values tabulated for *m* evenly spaced intervals of size Δ*r* = *r*_cut_/(*m* − 2) along the separation distance *r*_*i*_ = *i* × Δ*r* with the cut-off *r*_cut_, and *t* is given by

$$\begin{array}{c} t=\frac{r-r_{k}}{\Delta r}, \end{array}$$ (3.2)

where index *k* is determined such that *r*_*k*_ ≤ *r* < *r*_*k*+1_. We choose CBSPL form because it exhibits remarkable flexibility, and it can represent various complex functional characteristics of pair potentials for sufficiently large number of knots.

To ensure the stability of the relative entropy minimization, some precautionary measures are taken. For the Newton-Raphson update to converge towards a minimum, the Hessian, **H**, must be positive definite at each step. With a good initial guess for the CG parameters and by adjusting the value of the relaxation parameter, *χ*, stability of the Newton-Raphson method can be ensured. One approach to initialize the CG parameters can be to fit them to PMF obtained by inverting the pair distributions of the CG sites obtained from the reference AA ensemble. For the CBSPL form, which is linear in it’s parameters, the second derivative of *S*_rel_ is never negative, hence the minimization converges to a single global minimum. However, due to locality property of the CBSPL form, i.e., update to *c*_*i*_ affects only the value of the potential near *r*_*i*_, and the poor sampling of the very small separation distances in the high repulsive core, the rows of **H** corresponding to the first few spline knots in the repulsive core may become zero causing **H** to be a singular matrix. To avoid this singularity issue, we specify a minimum separation distance, *r*_min_, for each CG pair interaction and remove the spline knots corresponding to the *r* ≤ *r*_min_ region from the Newton-Raphson update. Once the remaining knot values are updated, the knot values in the poorly sampled region, i.e., *r* ≤ *r*_min_, can be extrapolated. The value of *r*_min_ can be estimated from the minimum distance at which the reference CG pair distribution is nonzero. Also, to ensure that the CG pair potentials and forces go smoothly to zero near *r*_cut_, couple of knot values before and after *r*_cut_ are fixed to zero.

For the convergence check, we define two types of errors: (*i*) the CG parameter error, *ϵ*_*λ*_, given by

$$\begin{array}{c} \epsilon_{\lambda }^{k}=\sum_{i=0}^{n-1}{\left(\lambda_{i}^{k}-\lambda_{i}^{k-1}\right)}^{2}, \end{array}$$ (3.3)

where *n* is the total number of CG parameters to be optimized, *k* is the index of the iteration step and (*ii*) the CG potential error, *ϵ*_*u*_, given by

$$\begin{array}{c} \epsilon_{u}^{k}=\sum_{i=0}^{M-1}\sum_{j=0}^{N-1}{\left(u_{i}^{k}\left(r_{j}\right)-u_{i}^{k-1}\left(r_{j}\right)\right)}^{2}, \end{array}$$ (3.4)

where *M* is the number of CG pair potentials to be optimized, and *N* is the the number of discrete points used to model CG potentials. Then, the total error, *ϵ*_tot_, is defined as

$$\begin{array}{c} \epsilon_{\text{tot}}^{k}=w_{\lambda }\;\epsilon_{\lambda }^{k}+w_{u}\;\epsilon_{u}^{k}, \end{array}$$ (3.5)

where *w*_*λ*_ and *w*_*u*_ are the weights assigned to the CG parameter error and the CG potential error, respectively. Iterations are terminated when *ϵ*_tot_ is less than the specified tolerance value or the specified limit of maximum number of iterations is reached. Finally, due to stochastic nature of the CG simulations, optimal parameters are computed by evaluating their average over the last few iterations.

### 3.3 Computational cost

In the simplex and relative entropy algorithms, CG-MD simulations and analysis of the CG ensemble configurations at each iteration step are the main computationally expensive stages. The costs of the CG-MD simulation and analysis of the CG ensemble configurations increase with the size of the system, i.e., number of particles, and the length of the simulation, i.e., number of time steps. To ensure the convergence of the coarse-graining iterations, statistically reliable CG ensemble configurations are required, which can be obtained by selecting sufficiently large system size and long simulation length. An optimal system size and simulation length to reduce the cost of coarse-graining can be determined by doing few trial iterations.

In VOTCA, to perform coarse-graining in a computationally efficient manner, we use external MD simulation packages, such as GROMACS, LAMMPS, etc. (see Sec. 3.5), which are specifically developed for efficient MD simulations. For efficiently analyzing CG configurations, we implement parallel analysis framework in VOTCA, which is explained in the following subsection 3.4.

### 3.4 Parallel Analysis

Analysis routines, such as the calculation of radial distribution functions, can be computationally expensive. In fact, post-processing of MD simulation data may take as much time as the actual simulation itself. Usually, the cost of an analysis increases with the number of particles in the system as well as the number of time steps. In the previous serial versions of VOTCA, an analysis is performed in the sequential order on a frame-by-frame basis. In this work, we implement a thread-based parallel framework in order to speed up the post-processing of MD simulation data by making use of the multi-core architecture of modern processors.

The thread-based parallelization is implemented using a straight-forward embarrassingly parallel approach. Post-processing of trajectories is usually performed by applying the same calculations independently at all time steps. Therefore, straight-forward parallelization can be achieved by dividing a trajectory into time intervals, i.e., frames, and distributing these among cores or threads. In contrast, another possibilty would be to divide of a single frame into chunks, such as domain-decomposition schemes per frame, followed by the distribution of these chunks to cores. A domain-decomposition per frame based parallelization, although not applicable to all algorithms, would lead to a performance benefit independent of the number of frames to process. For the thread-based parallelization of the VOTCA analysis tools, we use the *POSIX* thread library. A thread-based implementation offers the advantage of shared-memory architectures, where memory can be accessed from all working nodes directly. The evaluation of each frame is now distributed among threads. For this purpose, the distribution or forking of the trajectory among threads, as well as the merging of the data from different threads has to be defined. However, the analysis part of the code remains unchanged as in the serial version. Single frames are then processed individually by each thread. By default, frames are evaluated according to their original order and output also follows the same order. Mutexes are used to impose the correct order of the frames and to handle locking of the input and output routines. A mutex, short for mutual exclusion, is typically used to serialize data access, where the parallel access of concurrent threads would lead to an undefined behavior. A mutex allows only one thread to access a block of code, while other threads have to wait until that first thread finishes this part of the code. The implementation, due to its simplicity, does not require the user to know much about parallel concepts. Most analysis algorithms can be parallelized with little code modifications, such as adjusting forking and merging details.

### 3.5 Additional Improvements

In addition to the new methods, we added support for coarse-graining bonded interactions by IBI [23], an extension to the IBI process to refine the potential to match the Kirkwood-Buff integrals [19], and iterative refinement of the thermodynamic force [24], which is needed for adaptive resolution simulations [25]. In addition to the technical improvements, we added interfaces to LAMMPS [26], Espresso [27], Espresso++ [28], HOOMD-blue [29] and DLPOLY [30], to complement the existing GROMACS [31] interface, which was updated to support GROMACS 5.0 [32]. This approach is a major advantage over other coarse-graining packages [33, 34], where an internal, very specialized, MD or MC engine is used for sampling and hence, often has limited simulation capabilities in comparison with the other very general MD codes mentioned above. Minor changes in CG mapper have been implemented to ensure compatibility with the STOCK package [35]. Last but not least, we also added the support for using hybrid methods, like a combination of FM and Boltzmann inversion [17].

## 4 Results

To illustrate functionality of the downhill simplex and relative entropy-based coarse-graining methods implemented in the VOTCA package, we have coarse-grained two systems: an SPC/E [36] bulk water system and a system consisting of a methanol-water mixture. In previous work, Mashayak and Aluru [37] have already used the relative entropy method in its VOTCA implementation to determine CG potentials for water confined inside of graphene slit channels at different thermodynamic states.

### 4.1 Coarse-graining of bulk water

In the past, water has already been studied extensively from the point of view of both all-atom and coarse-grained representations [8, 9, 13, 38–41]. CG potentials for bulk water obtained by IBI, IMC, and FM methods using the VOTCA package can be found in ref. [16]. In this work, we coarse-grain SPC/E bulk water at a thermodynamic state of 300 K temperature and 1 bar pressure using the simplex and relative entropy methods. In the coarse-grained model, we represent one water molecule by one CG bead positioned at its center of mass (COM), such that the CG beads solely interact via an isotropic two-body potential. Note, that representing multiple water molecules within one CG bead is also possible [42, 43]. Previously, Shell [12] and Chaimovich and Shell [44] have already employed the relative entropy method to optimize CG potentials for SPC/E water at various thermodynamic states, providing an appropriate reference for comparison. Also, recently Lu et al. [45] derived a series of coarse-grained potentials for various water models, TIP3P, SPC/E, TIP4P-Ew, and TIP4P/2005, using the relative entropy method and systematically compared the ability of these CG potentials to reproduce various structural, dynamic, and thermodynamic properties of water.

For the reference all-atom ensemble, we use the same bulk water all-atom configurations as in the NVT ensemble generated in ref. [16]. The all-atom system consisted of 2180 water molecules in a cubic box of size 4.031 nm. The coarse-grained system consisted of 2180 CG beads which are obtained by mapping the all-atom configuration using a COM mapping scheme.

For the simplex method, we use a modified Lennard-Jones (LJ) potential [46] as the functional form for the CG water interactions:

$$\begin{array}{c} U_{\text{CKD}}\left(r\right)=\{\begin{array}{cc} 4\epsilon (\frac{\sigma }{r^{12}}-\frac{\sigma }{r^{6}}+\frac{1}{4}) & :r<r_{\text{c,LJ}}\\ -\epsilon (\cos^{2}(\frac{\pi (r-r_{\text{c,LJ}})}{2w_{\text{c}}}) & :r_{\text{c,LJ}}<r<r_{\text{c,LJ}}+w_{\text{c}}\\ 0 & :r_{\text{c,LJ}}+w_{\text{c}}<r \end{array}, \end{array}$$ (4.1)

with an additional Gaussian,

$$\begin{array}{c} U_{\text{CKDg}}\left(r\right)=U_{\text{CKD}}\left(r\right)+h{\text{e}}^{\frac{-{\left(r-p\right)}^{2}}{2s^{2}}}-h{\text{e}}^{\frac{-{\left(r_{\text{c}}-p\right)}^{2}}{2s^{2}}}. \end{array}$$ (4.2)

In Eqs (4.1) and (4.2), *σ*, *ϵ* and *r*_c,LJ_ are the usual LJ parameters, *w*_c_ is the smoothness parameter for the attractive part of the CKD potential, and *h*, *p*, *s*, and *r*_c_ are the gaussian’s amplitude, mean, width, and cutoff radius, respectively. Hence, there are a total of 6 parameters, i.e., *σ*, *ϵ*, *w*_c_, *h*, *p*, and *s*, to optimize. We choose the modified LJ plus Gaussian form because a previous study [16] implied that the water-water CG interaction requires two potential energy minima. Initial parameters are obtained by fitting to the potential from ref. [16] and varying them slightly to obtain 7 sets of initial parameters.

We determine 2 sets of parameters for the CKDg functional form in Eq (4.2). The first set of the CKDg parameters is determined by using the reference AA ensemble water-water COM radial distribution as a target property. The second set of the CKDg parameters is determined by attempting to reproduce the radial distribution as well as the pressure of the AA ensemble. For the simplex algorithm, the penalty value, *y*, is defined as

$$\begin{array}{c} y=\sum_{r_{i}=0.0}^{0.6}|g_{\text{CG}}\left(r_{i}\right)-g_{\text{AA}}\left(r_{i}\right)|+a|p_{\text{CG}}-p_{\text{AA}}|, \end{array}$$ (4.3)

where *g*_CG_(*r*_*i*_) and *g*_AA_(*r*_*i*_) are the CG and the target AA ensemble RDF, respectively, *r*_*i*_s are the bin positions used to determine RDFs, *p*_CG_ and *p*_AA_ are the CG and the target AA ensemble pressure values, respectively, and *a* is the tunable parameter used to switch between only the RDF-based and RDF+pressure-based coarse-graining. For the latter we used *a* = 0.01/bar. In all of the simplex iterations, the 200 ps CG simulations are started from the same initial configuration, but energy minimized before the actual sampling, neglecting the first 20 ps. The minimization is necessary due to the fact that in the case of an expansion move, the initial CG configurations can be far away from any stable structure.

Using the relative entropy method, we optimized the CBSPL functional form, Eq (3.1), for the water-water coarse-grained interaction. For the CBSPL form, we have used a cutoff distance of 0.9 nm, fixed the grid spacing to 0.02 nm, and set *r*_min_ = 0.24 nm, i.e., only the knot values corresponding to the region, *r* > *r*_min_, are optimized, and the knot values in the poorly sampled region, *r* ≤ *r*_min_, are extrapolated. Therefore, there are 48 CG parameters for the water-water CG potential. The initial guess for the CG potential parameters was obtained by a least-square fitting of the CBSPL functional form to the PMF obtained by inverting the water-water COM pair distribution function from the reference AA ensemble. At each iteration, a CG simulation of 200 ps is performed with the GROMACS simulation software. For the first iteration, the initial configuration for the CG simulation is obtained by mapping the last configuration of the reference AA ensemble trajectory. For all subsequent iterations, the final CG configuration from the previous step is used as an initial configuration for the CG simulation. Furthermore, at each iteration, CG configurations corresponding to the first 50 ps are discarded as equilibration stage and only the configurations of the last 150 ps stored at 1 ps intervals are used to compute the update for the CG potential parameters.

**Fig 1:**
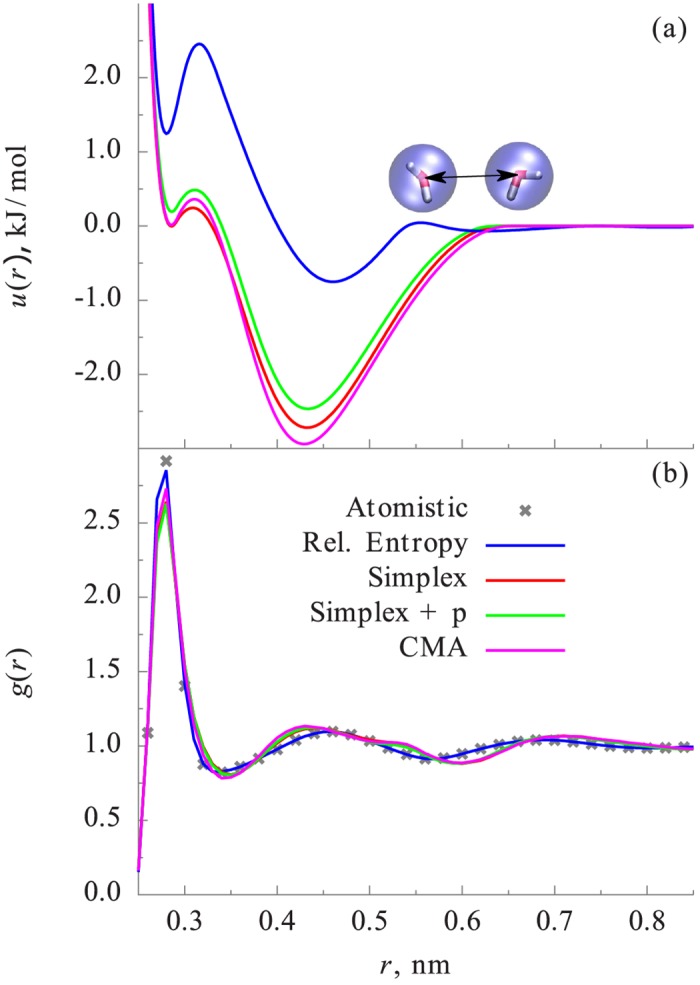
Comparisons of CG potentials (a) and RDFs (b) for bulk water. All methods fit the target RDF sufficiently well and lead to potential of similar shape. The relative entropy method has a slightly smaller error due the fact that more parameter are available in the potential form.

Fig 1a shows the optimized CG potentials for bulk water. It can be seen that, although the CG potentials from the relative entropy minimization and simplex are quantitatively different, they exhibit a similar core-softened double-well-type shape, which is a very characterisitc of the water-water CG potential [16, 44].

Fig 1b shows the water-water RDFs from the CG simulations along with the reference RDF from the AA simulation. One observes that the relative entropy-based CG potential is able to predict the water-water RDF accurately. The accuracy of the RDF from the relative entropy-based CG potential is consistent with the analysis made in ref. [14] and [15]. In these references, it is demonstrated that, when CG potentials are modeled using a finely tabulated functional form such as CBSPL, the relative entropy minimization would result in the CG potentials similar to IBI and IMC, which reproduce the target AA ensemble pair distributions. The simplex-based optimization approach is able to fit the radial distribution reasonably well considering the fact that there are only 6 parameters in contrast to the 48 parameters for the CBSPL form. Essentially simplex and CMA lead to the same result, which is not surprising for such a simple system where a local minimum is relatively easy to find. Fitting the RDF and pressure simultaneously leads to an interaction similar in shape to that of RDF-only, but shifted towards lower energies, which is in agreement with earlier findings [9].

### 4.2 Coarse-graining of water-methanol mixtures

To further test robustness of the simplex and relative entropy methods and their implementation within the VOTCA package, we consider a much more complex system, namely a water-methanol mixture. Methanol is the smallest alcohol and its structural properties feature a winding hydrogen-bonded chains with an average of approximately 2 hydrogen bonds per molecule [47]. Coarse-grained potentials for pure liquid methanol system have been determined by IBI, IMC, and FM methods in ref. [16]. In addition, a water-methanol mixture is a suitable system for studies of several structural aspects of solvation in aqueous mixtures [48].

In this study, we considered 3 different water-methanol mixtures with methanol mole fractions, *X*_*m*_, of 0.062 (diluted), 0.5 (equimolar), and 0.938 (concentrated), similar to the ones used in ref [48]. Reference AA simulations were performed in the NVT ensemble using the GROMACS simulation software [31]. Water is modeled using the SPC/E model, whereas the OPLS [49] force field was used to model methanol, and the LJ interaction parameters, namely *C*_12_ and *C*_6_, for the cross-interactions between water and methanol are determined using a geometric mean rule. The number of molecules and average densities of the simulated solutions are given in Table 1. All three mixtures were simulated in a cubic box of length 5.05691 nm with periodic boundary conditions, at 300 K temperature maintained using the Nosé-Hoover thermostat [50]. Systems were equilibrated for 5 ns each, followed by production runs of 20 ns. Reference radial distributions have been computed using snapshots at every 1 ps and a bin size of 0.01 nm.

**Table 1:** Simulated water-methanol mixtures.

	I	II	III
number of H_2_O	3752	2000	248
number of MeOH	248	2000	3752
*X*_*m*_	0.062	0.5	0.938
*ρ* g/cm^3^	0.97	0.885	0.80

In the CG model for the water-methanol mixtures, water and methanol molecules are represented by CG beads positioned at their COM and the interactions between CG beads are modeled via isotropic two-body potentials. The number of water and methanol molecules in the CG simulations are the same as in the reference AA simulations (see Table 1).

The simplex setup is very similar to the above mentioned bulk-water setup. We have used the CKDg potential (Eq (4.1)) to model the coarse-grained interactions, yielding a total of 18 parameters to optimize.

For the relative entropy-based coarse-graining, we have used the CBSPL form, Eq (3.1), to model all three CG interactions, i.e., water-water, water-methanol, and methanol-methanol interactions. For the water-water CG potential, a cut-off distance of 1.0 nm was used with the grid spacing of 0.01 nm and *r*_min_ = 0.24 nm. For the water-methanol and methanol-methanol CG potentials, a cut-off distance of 1.32 nm was used with the grid spacing of 0.02 nm. *r*_min_ for the water-methanol and methanol-methanol CG potentials was set to 0.27 and 0.3 nm, respectively. Therefore, there are total of 241 (103 for water-water, 69 for water-methanol and methanol-methanol) CG parameters to be optimized. At each iteration, a CG simulation of 500 ps is performed with the GROMACS and the CG configurations corresponding to the first 100 ps are discarded as equilibration stage and the configurations of the last 400 ps stored at 1 ps intervals are used to compute the update for the CG potential parameters.

**Fig 2:**
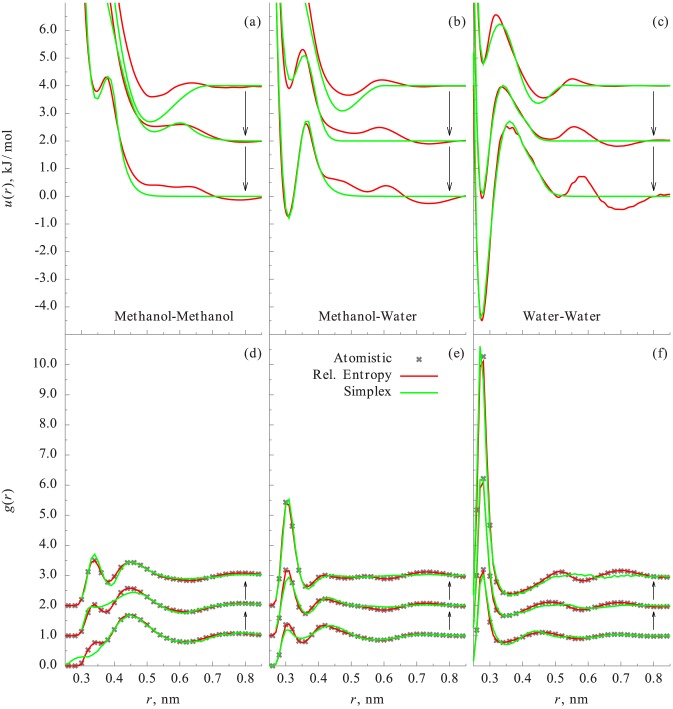
Comparisons of the CG potentials and RDFs of methanol-water: Mixture at different methanol mole fractions, *X* = 0.062,0.5,0.938 are shown. Arrow indicates the direction of increasing *X*. All methods fit the target RDF very well for all mole fractions.

The CG potentials obtained from the simplex optimization and the relative entropy minimization for the three different water-methanol mixtures are shown in Fig 2 along with the corresponding RDFs obtained from the CG simulations. Observations about the accuracies of the CG potentials from the simplex and relative entropy methods are similar to that of the bulk water case. Despite the limited flexibility of the CKDg form, the CG potentials from the simplex optimization are able to predict the RDFs reasonably well. As expected, due to the finely tabulated nature of the CBSPL functional form, the CG potentials from the relative entropy optimization are able to predict the water-water, water-methanol, and methanol-methanol RDFs as accurately as the reference AA simulations. We note that the CG potentials for the water-methanol mixture system are different for different mole-fractions. This is not surprising, because it is well-known that the CG potentials depend on the thermodynamic state of the reference system [8, 9]. However, it is possible to optimize CG potentials for multiple state-points simultaneously [51], but it is beyond the scope of this work.

### 4.3 Computational cost comparisons

Table 2 shows the comparison of the average computational cost per iteration step for the simplex and relative entropy-based coarse-graining of the water and water-methanol systems. Please note that, an iteration in VOTCA does not necessary correspond to an iteration in the classical definition of a simplex step. In VOTCA one iteration means running one coarse-grained simulation, while in the simplex method one iteration means one transformation of the polytope which can involve multiple coarse-grained simulations. The computational costs are obtained on a desktop machine with 8-cores Intel Xeon W3520 2.67GHz CPU. As described in the subsection 3.3, the CG-MD simulation and CG configuration analysis are the two major computational parts in the simplex and relative entropy-based coarse-graining. Therefore, in Table 2, we show the distribution of the total computational cost into the CG-MD and CG analysis parts. We observe that, for the systems studied in this work, the simplex is faster than the relative entropy, even though a energy minimization is performed in each simplex step, mainly because the CG analysis step of the relative entropy requires more time than the simplex. The CG analysis step of the relative entropy is more computationally expensive than the simplex due to two main reasons: (i) the number of CG parameters in the relative entropy coarse-graining is more than the simplex and (ii) for the relative entropy update of the CG parameters, in addition to processing CG configurations to evaluate the Hessian and the derivative matrices, we need to solve the system of linear equations, i.e., Eq (2.8), which requires 𝓞(*n* ^3^/3) computational cost when solved using the Cholesky decomposition. For the methanol-water systems, the CG-MD simulation lengths in the simplex and relative entropy-based coarse-graining are the same, and hence, the CG-MD cost is similar in both the methods. However, for the the bulk water case, the relative entropy CG-MD requires more time than the simplex, because the length of the CG-MD in the relative entropy is longer (100000 steps) than the simplex (500 steps). We also note that, for both the simplex and relative entropy methods, the computational cost of coarse-graining water-methanol systems is higher than the bulk water because of the higher number of CG parameters, the larger CG-MD system size (see Table 1), and longer CG-MD simulations at each iteration step.

**Table 2:** Average computational cost per iteration step (in seconds) for simplex and relative entropy-based coarse-graining. For the simplex methods the number in the bracket denote the time spend in energy minimization before the actual molecular dynamics part.

	Total	CG-MD	CG analysis
Water			
Simplex	16.3	4.3(+ 0.08)	11.92
RE	435	124.5	310.5
Water-methanol (X = 0.062)			
Simplex	2140.2	2109.9(+ 5.29)	25.01
RE	5076	2137.4	2938.6
Water-methanol (X = 0.5)			
Simplex	1555.2	1522.3(+ 0.76)	32.14
RE	4176.9	1631.8	2545.1
Water-methanol (X = 0.938)			
Simplex	1165.8	1130.2(+ 0.87)	34.73
RE	2796.7	1151.3	1645.4

### 4.4 Parallel Analysis

We demonstrate the performance of the parallel analysis implementation by calculating the RDF of a Lennard-Jones [52] fluid. Furthermore, we highlight a crucial part of many analysis algorithms: constructing neighbor lists, i.e., the search for the neighbors of a particle within a cut-off distance. Neighbor lists are often required in internal loops, such as the computation of the distance between two particles within a sphere. Depending on the analysis algorithm and system size, the construction of neighbor lists may dominate the total computational cost. We compare two approaches for constructing neighbor lists- (i) a naive approach of the simple search and (ii) the more efficient implementation based on the grid search. The performance of the analysis is measured as a function of the system size and neighbor search cut-off.

The simple search algorithm for constructing neighbor lists is as following. For each particle in a system, loop over all other particles and check the distance between the two particles. If the distance is less than the provided cut-off, tag this pair as neighbors. Therefore, the simple search algorithm requires to loop through all particles twice, and hence, its expected computational cost is 𝓞(*N* ^2^), where *N* is the number of particles.

In contrast, the grid search algorithm loops over all particles once and distributes the particles among a grid based on their positions. The size of the cells in the grid depends on the cut-off. A neighbor lookup will then lead to a constant cost of checking all surrounding cells. The grid search algorithm is expected to have, besides a large prefactor, a cost of 𝓞(*N*). The performance of the grid search decreases with an increasing cut-off, since each cell in the grid will hold more particles, whereas the cost of the simple search does not depend on the cut-off.

VOTCA supports both the neighbor list algorithms, and the user is given a choice to select one of them.

For the performance analysis, we study three different LJ systems with different sizes: the small system with 5324, the medium with 17687, and the big with 60132 fluid particles. The system temperature and density are set to T* = 0.73 and ρ* ≈ 0.9 in the reduced LJ units, respectively.

**Fig 3:**
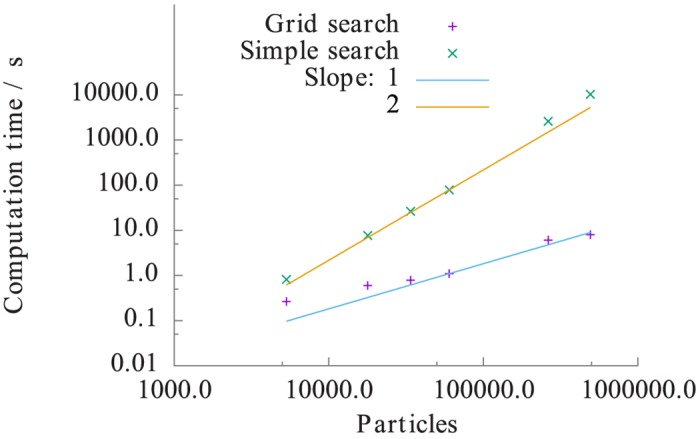
Computation times for the construction of neighbor lists: simple vs. grid search. Shown are the results for the Lennard-Jones fluid as a function of particles in the system. In addition to the data points for the simple and grid search algorithms, lines indicate the scaling law with 2 and 1 as the exponent, respectively. These exponents result from the cost of the simple and grid search algorithm: 𝓞(*N* ^2^) and 𝓞(*N*), respectively. The cut-off for the neighbor search was set to 1.6*σ*, which roughly corresponds to the first minimum in the radial distribution function.

First, we compare the performance of the simple search and grid search algorithms. For comparison, we measure the computational time for constructing neighbor-list for 1 frame on 1 core only. In Fig 3, the timing results for the neighbor-list creation as a function of the number of particles are shown. The computational times of the simple and grid search algorithms follow the expected scaling of 𝓞(*N* ^2^) and 𝓞(*N*), respectively. The grid search algorithm shows a very good agreement with our expectation. However, the simple search algorithm performs worse than expected for the systems with 262636 and 492038 particles. The performance loss of the simple search algorithm is likely due to the large amount of data the algorithm has to process in the inner loops. For large data, cache memory limits may exceed causing bad scaling. An additional benchmark, not reported here, confirmed the dependence of the grid search algorithm on the neighbor cut-off distance, whereas the simple search is unaffected. However, even after increasing the cut-off from 1.6 to 2.6*σ*, it is found that the grid search performs an order of magnitude faster than the simple search. The cross-over, where the simple search becomes faster than the grid search depends on the cut-off distance, but is typically too large for productive uses, and hence, the grid search should be preferred over the simple search in general.

Next, we study the overall performance of the parallel analysis framework. Ideally, we expect the speed-up of the parallel analysis, i.e., the ratio of the time required for the serial analysis to the time for parallel analysis, to match the number of threads used. Due to the use of shared-memory architecture, communication between threads is not necessary. Hence, the overall performance becomes more efficient with increasing intensity ratio, i.e., computational cost to input-output (IO) cost. However, the performance may be negatively affected due to the overhead of threads, such as the additional definition and initialization needed for the single thread and the handling of mutexes, as well as possible interferences while accessing the shared memory from multiple threads. Some of the mutex-overhead can be saved by explicitly neglecting the original ordering of frames. Both negative effects increase with the number of threads and reduce the overall speed-up.

**Fig 4:**
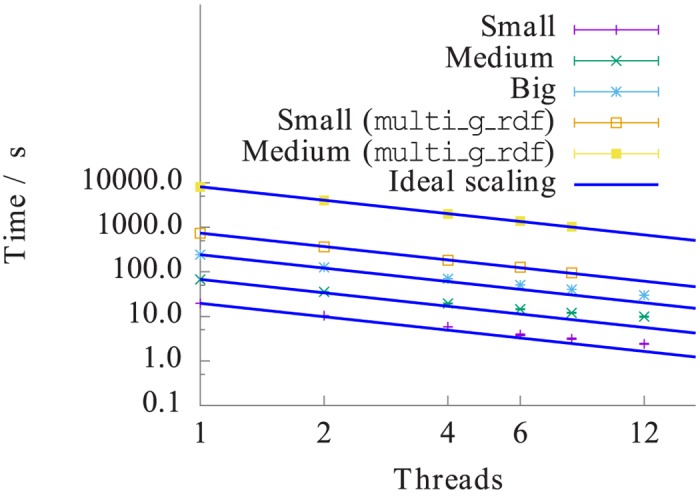
Absolute computation time for the radial distribution function calculation as a function of threads. The small system holds 5324, the medium 17687 and the big system 60132 particles. The dashed line shows the ideal scaling line. Also shown are the results from the script-based parallelization of multi_g_rdf.

Fig 4 summarizes the computational times for the calculation of *g*(*r*) as a function of the number of threads. It is observed that increasing the number of threads always decreases the total computation time, however, the timings deviate from the ideal scaling line with increasing thread count. Up to 4 threads, the computation time is closer to the ideal scaling, i.e, the parallel speed-up is around 3–4. For 6, 8, and 12 threads, however, the total computation time deviates from the ideal scaling line. The non-ideal time scaling behavior is mainly caused by the constant overhead of the VOTCA input routine and a missing cache optimization. The raw numbers of the computation time for all 960 frames on 1 core are 20s, 68s, and 243s for the small, medium and big system, respectively. For comparison, the GROMACS tool, g_rdf, which is based on simple search, takes 740s, 8116s and 82728s to compute *g*(*r*) for the small, medium and big system, respectively. In the former versions of VOTCA, the parallelization of g_rdf was script-based and handled by multi_g_rdf. The trajectory was explicitly split in time into chunks and multiple instances of g_rdf were called. The results for the small and medium systems are also shown in Fig 4.

As stated earlier, the overall performance gain is improved if computationally intensive algorithms are used. In other words, when using threads, a computationally expensive algorithm leads to a better scaling behavior. The constant overhead cost becomes more negligible and cache-misses are less likely to happen. For example, the algorithm that spends most of the CPU time in the inner loops of the evaluation, will perform much better than the lightweight calculation of a RDF. Our tests showed almost-perfect scaling up to 8 threads and only 20% loss of the performance at 12 threads for the algorithm which makes a heavy use of intrinsic CPU commands.

## 5 Conclusions

In this work, we have extended the versatility of the VOTCA package by implementing two recent coarse-graining methods, namely, the downhill simplex optimization and the relative entropy minimization. We have also demonstrated the applicability of the newly implemented coarse-graining techniques in VOTCA by coarse-graining a bulk water system and water-methanol mixture. We have found that for both the systems, the CG pair potentials described by the CBSPL functional form and optimized by minimizing the relative entropy reproduce the target pair distributions. This result validates the known characteristic of the relative entropy minimization, i.e., when the CG pair potentials are modeled using finely tabulated functional form then at relative entropy minimum the CG pair potentials reproduce the pair distributions of the target ensemble. We have also found that the simplex-based optimization method is effective in minimizing the error between the properties from the CG and the target AA simulations. For the bulk water and water-methanol mixtures, we have shown that the simplex method can effectively optimize the CG potentials modeled using the CKDg functional form with just 6 parameters, such that the target pair distributions and pressure values are predicted reasonably well by CG simulations. The accuracy of the CG potentials optimized by the simplex method can be improved if a more complex functional form with additional parameters is used to model the CG potentials. However, for a large number of parameters, the simplex method becomes too computationally inefficient. Input for these simulation will be made available as tutorials as part of the upcoming VOTCA 1.3. Finally, in addition to the new coarse-graining techniques, we have also improved the computational efficiency of the VOTCA package by implementing the parallel analysis framework and added support for more MD sampling engines.
